# Preparation and Photocatalytic Performance Study of TiO_2_-TMP Composites Under Effect of Crystal Structure Modulation

**DOI:** 10.3390/ma18112623

**Published:** 2025-06-03

**Authors:** Jiayi Zhang, Chen Wang, Xiaoguo Shi, Qing Feng, Tingting Shen

**Affiliations:** Division of Environmental Science & Engineering, Qilu University of Technology, Shandong Academy of Sciences, Jinan 250353, China; zjy15290555343@163.com (J.Z.); shixg@qlu.edu.cn (X.S.); qingfeng@qlu.edu.cn (Q.F.); shentingting@qlu.edu.cn (T.S.)

**Keywords:** titanium dioxide, crystalline structure, polymer, photocatalysis, heterojunction

## Abstract

Nano-titanium dioxide (TiO_2_) is currently the most widely studied photocatalyst. However, its rapid recombination of photogenerated carriers and narrow range of light absorption have limited its development. Crystal form regulation and polymer modification are important means for improving the photocatalytic activity of single-phase materials. In this paper, TiO_2_ materials of different crystal forms were prepared by changing the synthesis conditions, and they were compounded with trimesoyl chloride–melamine polymers (TMPs) by the hydrothermal synthesis method. Then, their photocatalytic performance was evaluated by degrading methylene blue (MB) under visible light. The mechanisms of influence of TiO_2_ crystal form on the photocatalytic activity of TiO_2_-TMP were explored by combining characterization and theoretical calculation. The results showed that the TiO_2_ crystal form, through interface interaction, the built-in electric field intensity of the heterojunction, and active sites, affected the interface charge separation and transfer, thereby influencing the photocatalytic activity of TiO_2_-TMP. In the 4T-TMP photocatalytic system, the degradation rate of MB was the highest. These studies provide theoretical support for understanding the structure–property relationship of the interfacial electronic coupling between TiO_2_ crystal forms and TMP, as well as for developing more efficient catalysts for pollutant degradation.

## 1. Introduction

Due to rapid industrialization, urbanization, and unlimited human activities, traditional fossil energy is consumed in large quantities [1,2]. The ever-growing energy demand has come into fierce conflict with the limited global fossil fuel reserves [3,4,5]. Developing sustainable energy resources and pollution control are currently the most urgent tasks for humanity [1,3,4]. Solar energy is the most abundant clean energy available [6,7]. Photocatalytic technology that uses renewable solar energy as a driving force for pollutant degradation offers a promising solution to energy shortages and severe global warming, which has aroused great interest in the scientific community [8,9]. However, industrial wastewater is complex in composition, especially considering refractory organic substances such as azo dyes and anthraquinone dyes discharged by the textile printing and dyeing industry [10]. Due to its environmental persistence, bioaccumulation, and potential carcinogenicity, it poses severe challenges to photocatalytic technology [11,12].

Under the current environmental situation, developing photocatalysts with high activity, low cost, and a good stability is the key to promoting the development of photocatalytic technology [3]. TiO_2_ is widely used in wastewater purification due to its non-toxicity, ease of preparation, and stable properties [13,14]. However, it can only respond to the ultraviolet light band, resulting in a low utilization rate of sunlight [15,16]. Moreover, defects such as a high recombination rate caused by photogenerated electron–holes seriously restrict its practical application efficiency [17]. The π electrons in conjugated polymers have a relatively high degree of delocalization [18,19]. When TiO_2_ is combined with them, the conjugated large π bond structure serves as the carrier transport channel, which can efficiently capture visible light and improve quantum yield [20,21].

In addition, the surface properties, band structures, electron migration characteristics, and atomic arrangement of TiO_2_ with different crystal forms are all different [22,23,24,25]. These differences further determine the photocatalytic performance of TiO_2_. The basic structural unit constituting TiO_2_ is the TiO_6_ octahedron [14,26]. The distortion degree of the TiO_6_ octahedron of titanium dioxide with different crystal forms affects its interaction with the surrounding components in the composite material, thereby changing the electronic properties of the composite catalyst [26]. Therefore, the photocatalytic activity of TiO_2_ is highly dependent on the crystal structure and the interfacial charge transfer efficiency. However, current research mostly focuses on the combination of a single-crystal form of TiO_2_ and polymers. A correlation analysis of the interface mechanisms between various crystal forms of TiO_2_ and polymers, especially the contribution of brookite-phase TiO_2_, has not been systematically explored. Moreover, previous studies have mostly focused on the phenomenon of improving the photocatalytic efficiency of composite materials, but lack in-depth analyses of its enhancement mechanisms (such as the charge transport path dependent on crystal form and the interaction between the conjugated polymer functional group and titanium dioxide crystal plane).

In this study, TiO_2_ of different crystal forms was synthesized by changing the reaction conditions and compounded with the trimesoyl chloride–melamine copolymer (TMP). Then, its photocatalytic performance was evaluated by degrading methylene blue (MB) under visible light. TMP was prepared by a previous group for the modification of titanium dioxide [27,28]. The influence law and mechanism of TiO_2_ crystal form on the photocatalytic activity of TiO_2_-TMP composites were explored through a combination of characterization and theoretical calculation. The structure–activity relationship of the interfacial electronic coupling between the TiO_2_ crystal form and TMP was revealed from the interfacial interaction, the built-in electric field strength of the heterojunction, and the active sites, providing new insights into the interface interaction mechanism of organic–inorganic composites. This is expected to promote and achieve the optimization of photocatalytic performance and develop more efficient catalysts for pollutant degradation.

## 2. Materials and Methods

### 2.1. Reagents

Trimesoyl chloride (TMC), melamine (MA), ethylenediaminetetraacetic acid disodium (EDTA-2Na), silver nitrate (AgNO_3_), and methyl alcohol (CH_4_O) were purchased from Shanghai Aladdin Chemistry Co., Ltd. Titanium trichloride (TiCl_3_), p-benzoquinone (p-BQ), and sodium nitrate (NaNO_3_) were obtained from Shanghai Maclean Biochemical Technology Co., Ltd. (Shanghai, China). Sodium hydroxide (NaOH), tartaric acid (C_4_H_6_O_6_), anhydrous ethanol (EtOH), and anhydrous copper chloride (CuCl_2_) were obtained from Sinopharm Chemical Reagent Co., Ltd. (Shanghai, China). All these reagents were all analytically pure, and all solutions were prepared with deionized water.

### 2.2. Preparation of Catalysts

**Figure 1:**
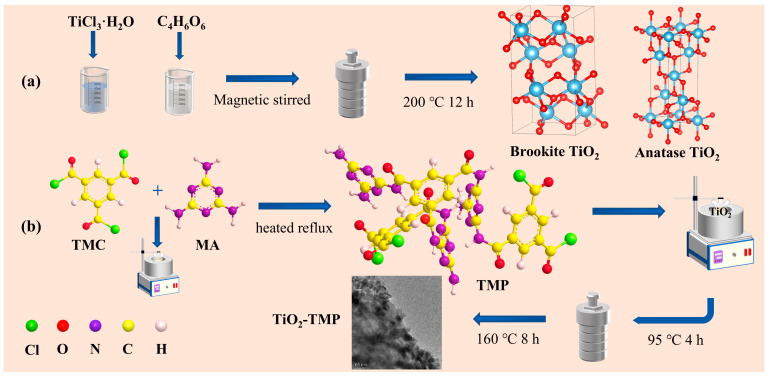
Schematic illustration of the material preparation.

For the preparation of TiO_2_, briefly, 5 mL of 15% TiCl_3_ solution was dissolved in a beaker containing 30 mL of deionized water. Subsequently, 0.276 g of NaNO_3_ was added to the above solution as an oxidizer and vigorously stirred for 30 min to form a colorless and transparent solution. Different amounts of C_4_H_6_O_6_ were added and stirred well. Then, 2 mol L^−1^ of NaOH aqueous solution was used to adjust the pH to 10. Afterwards, the mixture solution was injected into a 100 mL Teflon-lined stainless-steel autoclave and reacted at 200 °C for 24 h. After reactor cooling to room temperature, the obtained solids were collected by centrifugation with water and ethanol, and then dried in vacuum at 60 °C for 12 h. A series of samples were obtained by changing the molar ratio of C_4_H_6_O_6_ and TiCl_3_ (C_4_H_6_O_6_: TiCl_3_ = 0.00, 0.375, 0.5, and 0.75), recorded as 1T, 2T, 3T, and 4T (Figure 1). The synthesis process refers to the work of Shen et al. [29,30].

**Preparation of TMP.** The preparation method of TMP was detailed in our previous study [27]. A total of 2.54 g of trimesoyl chloride was placed in a 250 mL three-necked flask. Then, 5.35 g of melamine was quickly transferred to the reaction system, and 0.1 g of CuCl_2_ catalyst was added simultaneously. Then, the reaction device was placed in a constant-temperature oil bath system and heated to 95 °C. After stirring and reacting for 4 h, the obtained solids were collected by centrifugation with water and ethanol. Finally, they were dried in vacuum at 60 °C for 12 h (Figure 1).

**Preparation of TiO_2_-TMP composite catalysts.** TiO_2_-TMP was prepared according to previous reports, with some modifications [27]. The obtained TMP was dispersed in 100 mL of ethyl alcohol for 2 h by ultrasonication. The TMP solution was then dripped into a three-necked flask containing TiO_2_ nanoparticles. After stirring and reacting at 95 °C for 4 h, the mixture solution was transferred into a 100 mL Teflon-lined stainless-steel autoclave and reacted at 160 °C for 8 h. The obtained solids were collected by centrifugation with water and ethanol, and then dried in vacuum at 105 °C for 2 h. The entire preparation process of the catalysts is illustrated in Figure 1.

### 2.3. Characterization

An X-ray diffractometer (XRD, SMARTLAB SE, Japan) was employed using Cu Kα radiation (λ = 1.5406 Å) to characterize the crystal phase structure, relative crystallinity, and crystal size of the catalysts within the scanning range of 10–80°. The nitrogen adsorption–desorption isotherm of the catalysts was measured at 77 K using Autosorb iQ analyzer (Quantachrome Instruments, Graz, Austria). The mean pore sizes were obtained according to the Barrett–Joyner–Halenda (BJH) method, and the specific surface areas were calculated in terms of the standard Brunauer–Emmett–Teller (BET) model. The morphology and microstructure of the catalysts were observed by scanning electron microscopy (SEM, ZEISS G500, Oberkochen, Germany), transmission electron microscopy (TEM, JEM-14000F, Amagasaki, Japan), and high-resolution transmission electron microscopy (HRTEM, FEI, TECNAI G2 F20, Waltham, MA, USA). The distribution of relevant elements was analyzed using an energy dispersive X-ray spectrometer (EDS, FEI, TECNAI G2 F20, Waltham, MA, USA). In the wavelength range from 400 to 4000 cm^−1^, the chemical structure of the materials was characterized at room temperature by a Fourier transform infrared spectrometer (FT-IR, IRAffinity-1s, Shimadzu, Kyoto, Japan) using KBr as a background. X-ray photoelectron spectroscopy (XPS, K-Alpha, Thermo Fisher, Waltham, MA, USA) and VB-XPS were performed using a monochromatic Al Kα source at 20 kV. The photocatalytic activity and band gap values of the samples were analyzed using a UV–Vis diffuse reflection spectrometer (UV-3600Plus, Shimadzu, Kyoto, Japan). The work function of the samples was determined by an ultraviolet photoelectron spectrometer (UPS, ESCALAB 250XI, Thermo Fisher, Waltham, MA, USA). The active species •O_2_^−^ and •OH in the photocatalytic activity were detected using an electron spin resonance spectrometer (ESR, Bruker EMX PLUS, Ettlingen, Germany), with 5,5-dimethyl-1-pyrroline N-oxide (DMPO) as a spin trapping reagent.

Detailed calculation methods for the built-in electric field strength (BIEF) of the heterojunction are listed in Text S1.

The experimental contents of the electrochemical impedance spectroscopy, transient photocurrent density, and cyclic voltammetry are listed in Text S2.

### 2.4. Activity Test of Photocatalytic Degradation of Methylene Blue (MB)

The photocatalytic activity of the samples was tested by the photocatalytic degradation of methylene blue (MB) in water. A 300 W xenon lamp was used as the light source, and a 420 nm filter was used to simulate visible light (λ > 420 nm). In total, 0.1 g of catalyst and 150 mL of MB solution were added to the reactor and stirred for 30 min under dark conditions to achieve an adsorption–desorption equilibrium between the catalyst and reactants. Then, the reactor was irradiated with a xenon lamp and continuously stirred for 1.5 h, and during the light period, the reactor was placed in a cooling water circulation system. A total of 5 mL of solution was taken from the reactor every 10 min, filtered through a needle filter (0.22 µm), and the absorbance value of the solution at 514 nm was measured by UV–VIS spectrophotometer. The degradation efficiency of MB can be calculated by Formula (1).

η = ((C_0_ − C_t_)/C_0_) × 100% = ((A_0_ − A_t_)/A_0_) × 100% (1)

where C_0_ and C_t_, respectively, represent the initial concentration of MB and the concentration of MB at time t. A_0_ and A_t_, respectively, represent the initial absorbance of MB and the absorbance of MB at time t.

Details of the free radical trapping experiments are listed in Text S3.

### 2.5. Theoretical Calculation Methods

Details of the methods and parameters related to the theoretical calculations can be found in the Supporting Information (Text S4).

## 3. Results

### 3.1. Analysis of Interface Interaction Between TiO_2_ and TMP

#### 3.1.1. XRD Analysis

**Figure 2:**
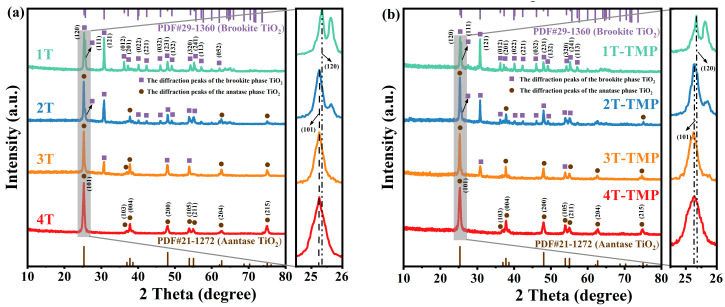
XRD patterns of (**a**) TiO_2_ and (**b**) TiO_2_-TMP.

XRD patterns were used to analyze the crystal phase structures of TiO_2_ and TiO_2_-TMP. As shown in Figure 2a, the synthesized TiO_2_ could be labeled as brookite (JCPDS 29-1360) and anatase (JCPDS 21-1272) phases. The phase composition (Table S1, see the Supplementary Materials) of all samples was estimated based on the integral intensities of reflections from anatase (101) and brookite (121) [31]. Firstly, when C_4_H_6_O_6_ was not added to the reaction system, the pure brookite-phase sample 1T was obtained. The XRD pattern revealed that the diffraction peaks of 1T at 2θ = 25.34° and 30.81° corresponded to the (120) and (121) crystal planes of the brookite phase, respectively. When C_4_H_6_O_6_ was added to the system, the anatase phase appeared in the prepared sample. With an increase in C_4_H_6_O_6_ dosage, the content of anatase phase increased, following the sequence of 2T, 3T, and 4T. Compared with TiO_2_, the characteristic diffraction peak positions of the TiO_2_-TMP composites did not change (Figure 2b), implying that the introduction of TMP had no significant influence on the crystal phase of TiO_2_. The crystallite size (Table S1) of the samples was calculated using the Debye–Scherrer formula [32]. The particle size after the combination of TiO_2_ with different crystal phases and TMP was decreased compared with that of TiO_2_, indicating that the modification with TMP effectively inhibited the crystal size of TiO_2_ [27]. Furthermore, the particle size of TiO_2_ decreased with an increase in anatase content. A small particle size of TiO_2_ could increase its contact area with TMP, enhance interfacial contact, and accelerate the interfacial transfer of electrons and holes.

#### 3.1.2. BET Analysis

**Figure 3:**
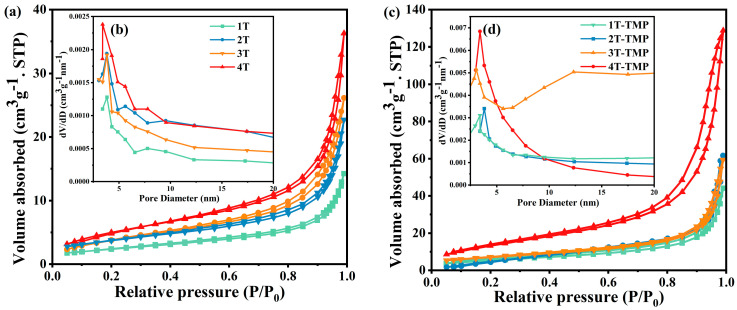
(**a**) N_2_ adsorption–desorption isotherms of TiO_2_; (**b**) pore size distribution curves of TiO_2_; (**c**) N_2_ adsorption–desorption isotherms of TiO_2_-TMP; and (**d**) pore size distribution curves of TiO_2_-TMP.

The N_2_ adsorption isotherms and pore size distributions of the TiO_2_ and TiO_2_-TMP composites are given in Figure 3. All samples exhibited a typical Langmuir IV isotherm. The H3-type hysteresis ring indicated that the material had a mesoporous structure [33]. Table S2 lists the specific surface areas and pore volumes of TiO_2_ and TiO_2_-TMP. Through comparison, it was found that the specific surface area of TiO_2_-TMP was higher than that of the pure TiO_2_, indicating that a more abundant pore structure was formed inside the composite material. Previous studies confirmed that the abundant pore structure in photocatalysts facilitates the exposure of more reactive sites, promoting the diffusion of products during the adsorption and degradation of pollutants, thereby enhancing the photocatalytic activity [34]. Notably, with an increase in the anatase phase content, the specific surface area of TiO_2_ increased from 8.558 m^2^g^−1^ (1T) to 13.464 m^2^g^−1^ (4T). A larger specific surface area can enhance the interfacial contact of TiO_2_ and TMP.

#### 3.1.3. SEM and TEM Images Analysis

**Figure 4:**
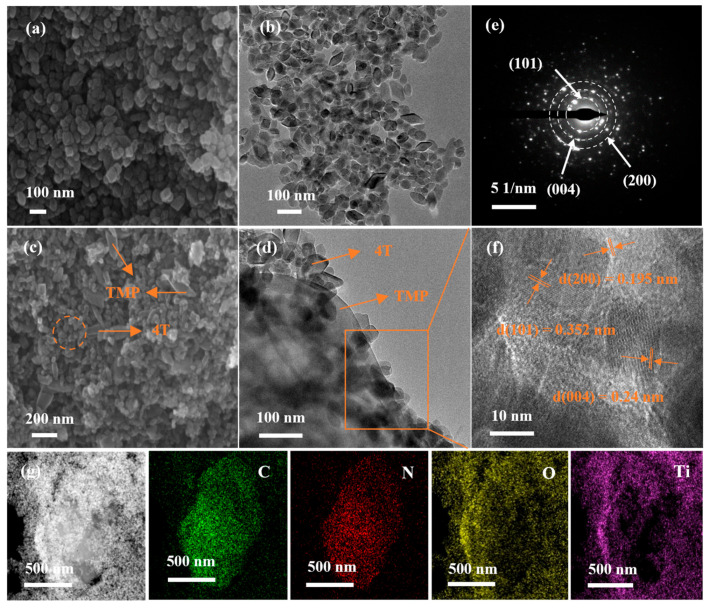
(**a**) SEM and (**b**) TEM images of 4T; (**c**) SEM and (**d**) TEM images of 4T-TMP; (**e**) SAED patterns of 4T-TMP; (**f**) HRTEM images of 4T-TMP; and (**g**) EDS mapping images of 4T-TMP.

The interface morphology and microstructure of TiO_2_ and TiO_2_-TMP were characterized by SEM, TEM, and HRTEM images. As shown in Figure 4a,b, TiO_2_ was a uniformly distributed rhombic structure. Figure 4c reveals a diamond-shaped structure of TiO_2_ nanoparticles attached on the smooth surface flake structure. Meanwhile, the irregular dark regions and overlapping bright zones in the TEM image of Figure 4d represent TiO_2_ nanoparticles and TMP, indicating sufficient interfacial contact between TiO_2_ and TMP, thereby forming effective heterojunction interfaces. Some obvious lattice fringes can be observed in the HRTEM image of 4T-TMP, and the lattice spacings of 0.195 nm, 0.24 nm and 0.352 nm correspond, respectively, to the (004), (200), and (101) crystal planes of anatase TiO_2_ (Figure 4f). Furthermore, the selected area electron diffraction (SAED) patterns in Figure 4e further confirm the successful synthesis of TiO2-TMP. As illustrated in Figure S1a,b, TiO_2_ was a uniformly distributed rod-like structure. The SEM image of 1T-TMP (Figure S1c) shows a rod-shaped structure of TiO_2_ nanoparticles attached on the smooth surface flake sheet-like structure. High-resolution TEM images reveal a lattice spacing of 0.29 nm for TiO_2_ (Figure S1e), which matches the (121) crystal plane of brookite TiO_2_. In addition, EDS mapping images show that the Ti and N elements overlap well at the interface in 4T-TMP, while the Ti element is sparsely distributed in the internal area in 1T-TMP (Figure 4g and Figure S1g, Supplementary Materials). The above results show that with an increase in the anatase phase content, the overlap between Ti and N elements at the interface intensifies. Therefore, the exposed crystal planes of TiO_2_ affected the interfacial contact between TiO_2_ and TMP.

#### 3.1.4. FT-IR Analysis

**Figure 5:**
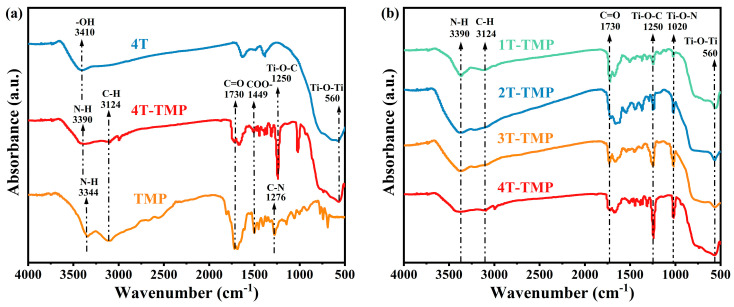
(**a**) FTIR spectra of 4T, 4T-TMP, and TMP and (**b**) FTIR spectra of TiO_2_-TMP.

The surface chemical structures of TiO_2_-TMP composites were analyzed by FT-IR. As shown in Figure 5, the absorption peaks of TiO_2_ near 560 cm^−1^ and 3390 cm^−1^ were the stretching vibration peaks of Ti-O and O-H, respectively [35]. The characteristic peaks of TiO_2_-TMP composites at 3390 cm^−1^, 3124 cm^−1^, 1730 cm^−1^, and 1449 cm^−1^ were, respectively, related to N-H, C-H, C=O, and COO- stretching vibrations [36], which were consistent with the peaks observed on TMP, but with significantly reduced peak intensities. Notably, two new absorption peaks emerged at 1250 cm^−1^ and 1020 cm^−1^ in TiO_2_-TMP, which were attributed to the stretching vibrations of Ti-O-C and Ti-O-N bonds, respectively [15,27]. These results demonstrate that TiO_2_ and TMP were not mechanically mixed, and it might be that the hydroxyl groups on the surface of TiO_2_ were chemically bonded to the C and N atoms on the carboxyl and amino groups in TMP. Furthermore, with an increase in the anatase phase content, the intensities of the Ti-O-C and Ti-O-N diffraction peaks increased progressively. Therefore, a strong chemical bond was formed between 4T and TMP. A tight combination of the interface is conducive to the migration of photogenerated carriers, thereby enhancing the photocatalytic activity [37,38]. It might be that the surface of anatase typically had more exposed OH^−^ prone to form Ti-O-N bonds with the N atoms in TMP (Figure S2) [39,40]. In addition, the strong chemical bonds acting as pillars promoted the expansion of interlayer spacing [41], which well explains that the 4T-TMP with the highest content of Ti-O-N bonds had the largest surface area and pore volume.

#### 3.1.5. XPS Analysis

**Figure 6:**
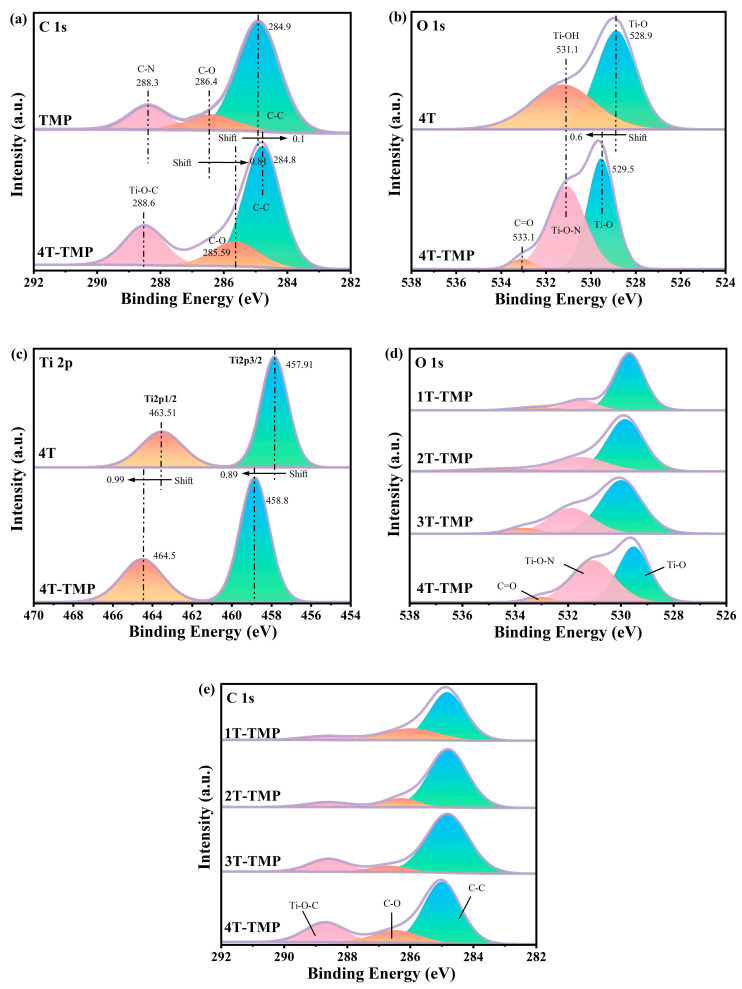
(**a**) XPS spectra of C 1s in TMP and 4T-TMP; (**b**) XPS spectra of O 1s in 4T and 4T-TMP; (**c**) XPS spectra of Ti 2p in 4T and 4T-TMP; (**d**) XPS spectra of O 1s in TiO_2_-TMP; and (**e**) XPS spectra of C 1s in TiO_2_-TMP.

To further investigate the internal chemical environment and bonding structure of TiO_2_-TMP, XPS spectra of C, O, and Ti elements were analyzed. It was found from the C 1s spectra (Figure 6a) of TMP and TiO_2_-TMP that a new characteristic peak emerged in TiO_2_-TMP, and the peak with a binding energy of 288.6 eV corresponded to the Ti-O-C bond [42,43]; The C 1 s spectrum of 4T-TMP could be deconvoluted into three peaks. The peak at 284.8 eV corresponded to uncertain carbon, the peak at 285.59 eV corresponded to the C-O bond, and the peak at 288.6 eV indicated the existence of a Ti-O-C bond [42,43,44]. As shown in the O 1s spectrum in Figure 6b, the characteristic peaks at 528.9 eV and 531.3 eV of 4T were ascribed to the Ti-O-Ti bond and Ti-OH bond formed by the oxygen of the surface OH^−^ [45,46,47]. The O 1s spectrum of the 4T-TMP composite material could be fitted as three peaks of 529.5 eV, 531.3 eV, and 533.1 eV, corresponding, respectively, to the stretching vibrations of the Ti-O, C=O, and Ti-O-N bonds [41,45,46,47]. As shown in the Ti 2p spectrum in Figure 6c, both 4T and 4T-TMP were deconvoluted into two peaks. Compared with 4T, the characteristic peaks of C 1s, O 1s, and Ti 2p of 4T-TMP shifted, which might be due to the formation of Ti-O-N and Ti-O-C bonds. When N forms a bond with Ti, the lone pair of electrons of N can transfer to the d orbital of Ti, increasing the electron density around Ti [48,49]. Therefore, the change in binding energy indicated that there was charge transfer at the TiO_2_ and TMP interface.

Notably, with an increase in the anatase phase content, the degrees of change in the binding energy of C 1s, O 1s, and Ti 2p in TiO_2_-TMP were enhanced (Figures S3–S5). The degree of binding energy shift determines electron coupling ability [41,50]. Moreover, the contents of the Ti-O-C bond and Ti-O-N bond also showed an obvious trend (Figure 6d,e), that is, 1T-TMP < 2T-TMP < 3T-TMP < 4T-TMP, with the specific proportions shown in Table S3. This is consistent with the FT-IR peak intensity results. The above results illustrate that TiO_2_ and TMP achieved tight interfacial coupling through chemical bond connection and provided a fast channel for charge transfer.

In summary, the particle size, specific surface area, crystal surface characteristics, and surface hydroxyl concentration of TiO_2_ with different crystal forms affected the interfacial contact between TiO_2_ and TMP and the content of Ti-O-N bonds. The interfacial contact between TiO_2_ and TMP was enhanced and the content of Ti-O-N bonds increased with an increase in the anatase phase content. The Ti-O-N bond provided an electron transport channel and affected the interfacial charge transfer. Therefore, the interaction between TiO_2_ and the TMP interface had a non-negligible influence on the photocatalytic performance of the composite materials.

### 3.2. Construction of Heterojunctions and Analysis of Energy Band Structures

The light responsiveness of the prepared samples was analyzed by UV–vis diffuse reflectance spectroscopy (DRS). As shown in Figure S6a, TiO_2_ had almost no absorption in the visible light region. Compared to pure TiO_2_, the spectral absorption edges of the TiO_2_-TMP composite material all underwent redshift, and the visible light absorption was enhanced. In addition, the band gaps of TiO_2_ and TiO_2_-TMP were calculated using the Kubelka–Munk Equation (2) [51], as follows:

αhν = A (Eg − hν) ^n/2^ (2)

where α is the absorption coefficient, h is the Planck constant, ν is the frequency of the incident photon, Eg is the band gap, and A is the absorbance. According to the properties of semiconductors themselves, both TiO_2_ and TMP are indirect band gap semiconductors (*n* = 4) [52,53]. The Eg values of 1T, 2T, 3T, and 4T were estimated to be 3.29 eV, 3.19 eV, 3.14 eV, and 3.15 eV, respectively, and the HOMO–LUMO band gap value of TMP was 3.72 eV (Figure S6b).

**Figure 7:**
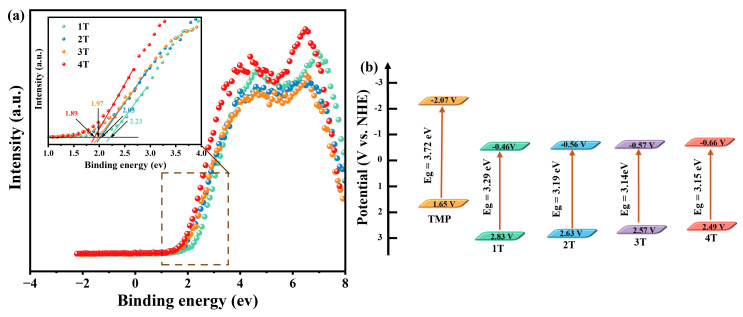
(**a**) The XPS valence band spectrum of TiO_2_ and (**b**) the band structure diagrams of TiO_2_ and TMP.

The valence band position (E_VB_) of TiO_2_ was obtained by the VB-XPS method [54]. As depicted in Figure 7a, the E_VB, XPS_ of 1T, 2T, 3T, and 4T were 2.23 eV, 2.03 eV, 1.97 eV, and 1.89 eV, respectively. The E_VB_ values of TiO_2_ were determined by Equation (3) [51,54,55], as follows:

E_VB_ = φ + E_VB, XPS_ − 4.44 (3)

Using Formula (3), the E_VB_ values of 1T, 2T, 3T, and 4T were further calculated as 2.83 V, 2.63 V, 2.57 V, and 2.49 V vs. NHE (the work function φ of the instrument is 5.04 eV). The HOMO energy level of TMP was determined to be 1.65 V vs. NHE by cyclic voltammetry (Figure S6c and Text S2). After that, the conduction band potentials (E_CB_) of TiO_2_ were determined by Equation (4) [56], as follows:

E_CB_ = E_VB_ − Eg (4)

The E_CB_ values of 1T, 2T, 3T, and 4T were calculated to be −0.46 V, −0.56 V, −0.57 V, and −0.66 V vs. NHE by Equation (4), respectively, and the LOMO energy level of TMP was −2.07 V vs. NHE. Based on these values, the band structure of the composite material could be constructed (Figure 7b). It can be seen from Figure 7b that there existed partial overlap between the energy bands of TiO_2_ and TMP, which might form Type II heterojunctions or S-scheme heterojunctions [57].

**Figure 8:**
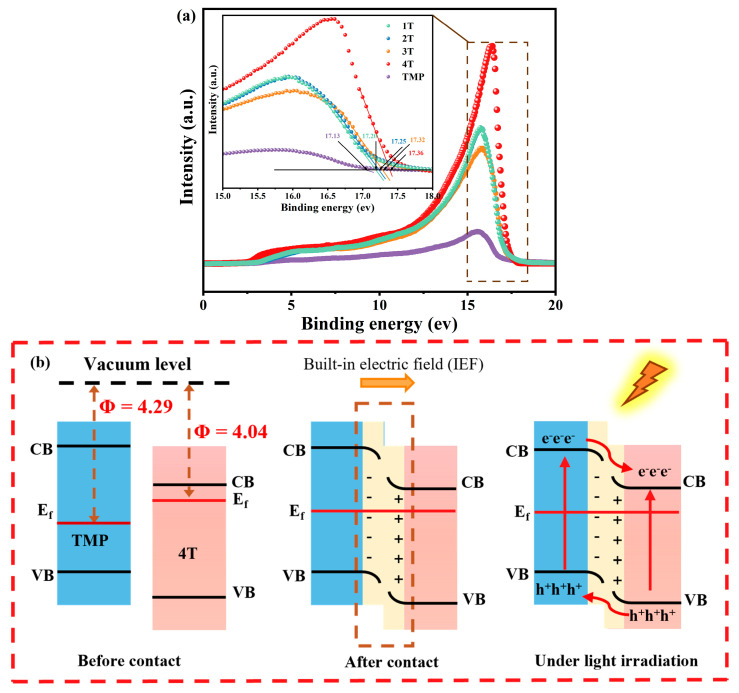
(**a**) UPS spectra of TiO_2_ and TMP and (**b**) the charge transfer mechanism diagram of TiO_2_-TMP heterojunction.

To confirm the type of TiO_2_-TMP heterojunction, the work function (Φ) was analyzed by ultraviolet photoelectron spectroscopy. The work function is the minimum energy required to eject electrons from the surface of a material, representing the electron loss ability of the material [58]. The work functions of TiO_2_ and TMP can be obtained through Equation (5) [59,60], as follows:

Φ = 21.22 eV − E_cutoff_ (5)

where 21.22 eV represents the excitation energy of He I, and E_cutoff_ refers to the secondary electron cutoff binding energy [59]. According to the E_cutoff_ values in Figure 8a, the Φ of 1T, 2T, 3T, 4T, and TMP were 4.02 eV, 3.97 eV, 3.92 eV, 3.86 eV, and 4.09 eV, respectively. The Fermi energy level (E_f_) can be defined by Equations (6) and (7) [60,61], as follows:

E_f (vs. AVS)_ = E_vac_ − Φ (6)

E_f (vs. NHE)_ = −4.44 − E_f (vs. AVS)_ (7)

where E_vac_ is the energy of the stationary electron at the vacuum energy level. Therefore, E_f_ was calculated as −4.02 eV (−0.42 V), −3.97 eV (−0.47 V), −3.92 eV (−0.52 V), −3.86 eV (−0.58 V), and −4.09 eV (−0.35 V) vs. AVS (vs. NHE). It was found through comparison that the Fermi energy level positions of TiO_2_ were all higher than those of TMP.

Furthermore, an increase in the binding energy of elements indicates a decrease in the electron density, meaning that atoms have lost electrons [62]. Therefore, a change in binding energy directly affects the migration direction of electrons in the heterojunction [57]. All the characteristic peaks of Ti 2p and O 1s in the TiO_2_-TMP heterojunction shifted toward the direction of an increasing binding energy, and all the characteristic peaks of C 1s shifted towards the direction of a reducing binding energy, indicating that during the formation of the heterojunction, electrons were transferred from TiO_2_ to TMP (Figure 6 and Figures S3–S5). Therefore, the type of TiO_2_-TMP heterojunction was determined.

As shown in Figure 8b, since the E_f_ of TiO_2_ was higher than that of TMP, after the close contact between TiO_2_ and TMP formed a heterojunction, the free electrons migrated from TiO_2_ to TMP to establish a Fermi energy level equilibrium, forming an interfacial built-in electric field (BIEF) from TiO_2_ to TMP, resulting in band bending. Thus, a Type II heterojunction electron transport path was formed [13,60,63,64]. This special electron transfer mode achieved the effective separation of photogenerated electron–hole pairs, thereby prolonging the lifetime of carriers [13].

**Figure 9:**
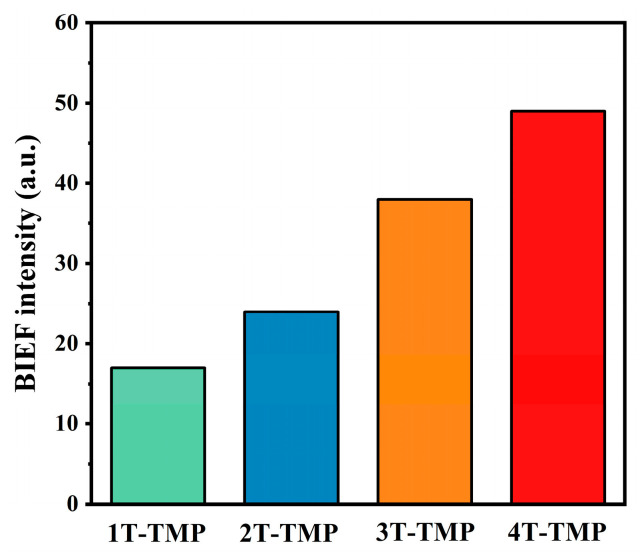
Built-in electric field (BIEF) intensity of TiO_2_-TMP.

The above discussions show that the band structure of TiO_2_ with different crystal forms affected the formation of TiO_2_-TMP heterojunctions. Firstly, the valence band and conduction band positions of 1T, 2T, 3T, and 4T were different. With an increase in the anatase phase content, the conduction band of TiO_2_ moved in a more negative direction (Figure 7b), which enhanced the generation of •O_2_^−^ and improved the photocatalytic degradation efficiency. Secondly, the Ef gap between TiO_2_ and TMP affected the intensity of the BIEF at the interface [60]. When the Ef gap between TiO_2_ and TMP expanded, the IEF of the TiO_2_-TMP heterojunction was enhanced, providing a higher driving force for the migration of photogenerated charges [65]. Therefore, we measured the BIEF intensity of the TiO_2_-TMP heterojunction (Figure 9 and Figure S7). The BIEF intensities of 1T-TMP, 2T-TMP, 3T-TMP, and 4T-TMP were 8.1, 10.3, 11.58, and 13.26, respectively. This fully demonstrates that the TiO_2_ crystal form affected the BIEF strength of the heterojunction due to the difference in band structure. When TiO_2_ was involved in the construction of heterojunctions, the promoting effect of anatase-phase TiO_2_ (4T) on heterojunctions was greater than that of plate titanite-phase TiO_2_ (1T). The intensity of BIEF directly affected the separation of photogenerated carriers and charge transfer in photocatalysts, thereby influencing their photocatalytic performance [64].

In summary, the interlaced distribution of the band structures and the difference in the Fermi energy levels of TiO_2_ and TMP formed Type II heterojunctions. The band structures of TiO_2_ with different crystal forms affected the formation of TiO_2_-TMP heterojunctions. The conduction band position of TiO_2_ affected the formation of •O_2_^−^, and the intensity of the heterojunction BIEF affected the separation and transfer of photogenerated carriers.

### 3.3. Research on Charge Transfer and Separation Mechanism

#### 3.3.1. Density Functional Theory (DFT) Calculation

**Figure 10:**
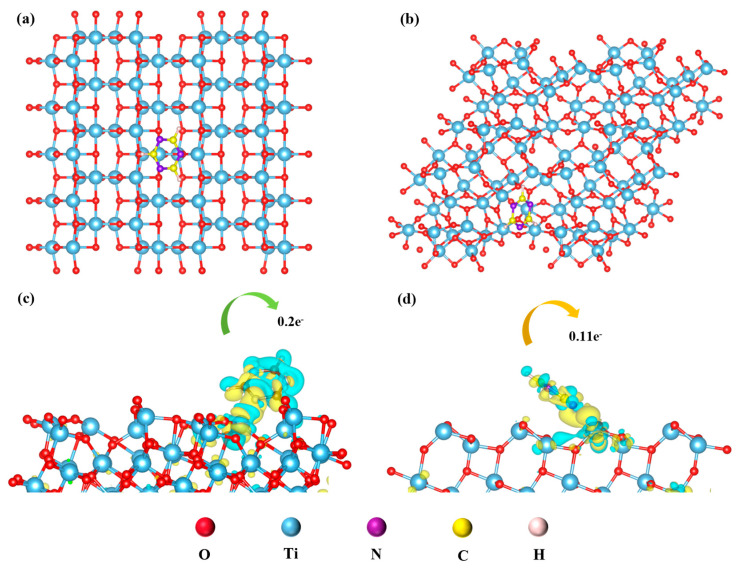
(**a**) Optimization model of anatase-phase TiO_2_ (101)-C_3_N_3_H_3_; (**b**) optimization model of brookite-phase TiO_2_ (121)-C_3_N_3_H_3_; (**c**) differential charge intensity map of anatase-phase TiO_2_ (101)-C_3_N_3_H_3_; and (**d**) differential charge intensity map of brookite-phase TiO_2_ (121)-C_3_N_3_H_3_.

Through DFT calculations, the influence of TiO_2_ crystal form on the charge transfer of TiO_2_-TMP and the interface charge transfer mechanism were further studied. The calculation methods and model construction are detailed in Table S3. Figure 10a,b show the structural optimization results of TiO_2_-C_3_N_3_H_3_. Based on this model, the differential charge density of TiO_2_-C_3_N_3_H_3_ was calculated. In the average charge density difference map (Figure 10c,d), we observed that the variation in electron density was concentrated at the interface, indicating that the Ti-O-N bond formed at the TiO_2_-C_3_N_3_H_3_ interface provided a channel for charge transfer. To quantify the variation in charge density, we conducted Bader charge analysis (Figure S8), where 0.2 and 0.11 electrons were transferred from TiO_2_ (101) and TiO_2_ (121) to C_3_N_3_H_3_, respectively. The electrons transferred to C_3_N_3_H_3_ in anatase TiO_2_ (101) were greater than those in brookite TiO_2_ (121), possibly because the Ti-O-N bond strength at the interface was higher. Furthermore, as shown in Table S4, the adsorption energy of the anatase (101) crystal plane for C_3_N_3_H_3_ was lower than that of the brookite (121) crystal plane, indicating a stronger adsorption capacity. Therefore, by constructing an adsorption model of the C_3_N_3_H_3_ and titanium dioxide, it was further demonstrated that the crystal form of TiO_2_ affected the interaction and charge transfer efficiency at the TiO_2_-TMP interface. Due to the low adsorption energy between anatase TiO_2_ and TMP, a stronger interfacial interaction might be exhibited; Furthermore, the number of electrons transferred from TiO_2_ of different crystal forms to TMP was different, resulting in different net charge accumulations. Net charge accumulation might affect BIEF intensity, thereby influencing the separation of photogenerated carriers and charge transfer [60].

**Figure 11:**
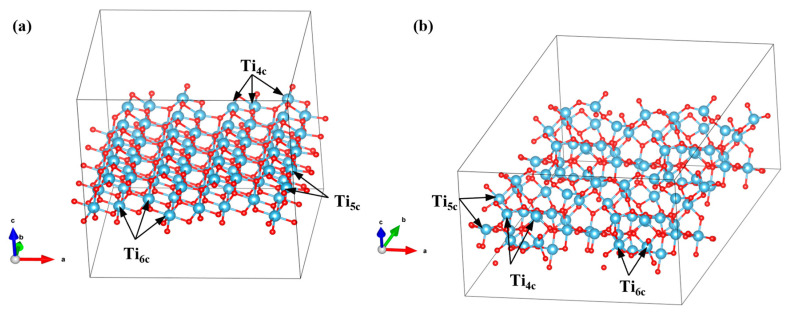
(**a**) The unit cell model of anatase-phase TiO_2_ (101) and (**b**) the unit cell model of brookite-phase TiO_2_ (121).

In order to better explain the influence of TiO_2_ crystal form on interfacial electron transfer and separation, the electronic structure and crystal plane characteristics of TiO_2_ were further analyzed. The projected density of states (PDOS) of TiO_2_ is shown in Figure S9. The density of states of Ti atoms near the Fermi level in the (101) crystal plane was higher than that in the (121) crystal plane. This means that the surface of anatase provided more available electronic states, which was conducive to the migration of electrons [23]. It could be found from the unit cell model that the atomic ratio of Ti_4c_: Ti_5c_: Ti_6c_ on the (101) crystal plane was 25%: 25%: 50% (Figure 11a). The atomic ratio of Ti_4c_: Ti_5c_: Ti_6c_ on the crystal surface of (121) was 12.5%: 75%: 12.5% (Figure 11b). There were more unsaturated coordination sites on the (121) crystal plan, which may have had more active sites. However, too many dangling bonds on the crystal plane of (121) might have led to structural relaxation, and the distorted structure caused the aggregation of Ti_4c_ sites. When TMP adsorbed, steric hindrance occurred, and the covering layer was uneven. The Ti_5c_ and Ti_4c_ sites on the crystal plane of (101) were uniformly distributed, which was conducive to the formation of dense interfaces by TMP molecules through chemical bonding. This explains that the Ti element and N element of 4T-TMP in EDS overlapped well at the interface. Therefore, the excellent electronic structure characteristics and atomic arrangement of the anatase (101) crystal plane can enhance photocatalytic activity to a certain extent.

#### 3.3.2. Photoelectric Performance Analysis

**Figure 12:**
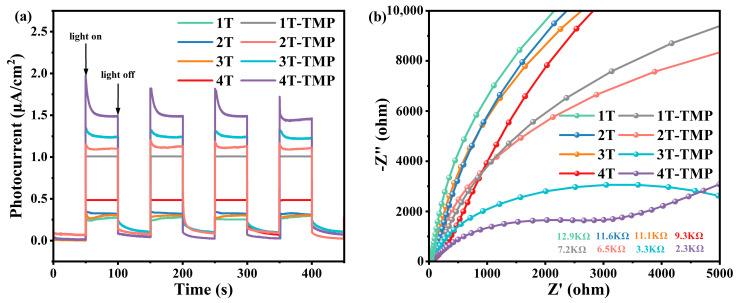
(**a**) Photocurrent response diagram of TiO_2_ and TiO_2_-TMP and (**b**) electrochemical impedance spectroscopy of TiO_2_ and TiO_2_-TMP.

The degree of photogenerated carrier separation and charge migration resistance of photocatalytic materials were evaluated by electrochemical impedance spectroscopy and transient photocurrent tests. As shown in Figure 12a, compared with TiO_2_, the photocurrent density of the composite material significantly increased; with an increase in the anatase phase content, the photocurrent density of the TiO_2_-TMP composite material gradually increased. This indicates that the construction of heterojunctions was conducive to improving the separation efficiency of photogenerated electron–hole pairs, and the TiO_2_ crystal form affected the interface charge separation of the TiO_2_-TMP composites. As shown in Figure 12b, compared with TiO_2_, TiO_2_-TMP had a smaller interfacial resistance; moreover, with an increase in the anatase phase content, the interfacial resistance of TiO_2_-TMP composites decreased. This indicates that the strong interaction between TiO_2_ and TMP could effectively reduce the interface resistance of the TiO_2_-TMP heterojunction, promote the migration of photogenerated carriers, and enhance photocatalytic activity.

### 3.4. Analysis of Photocatalytic Performance

#### 3.4.1. The TiO_2_-TMP Composites Degrade MB

**Figure 13:**
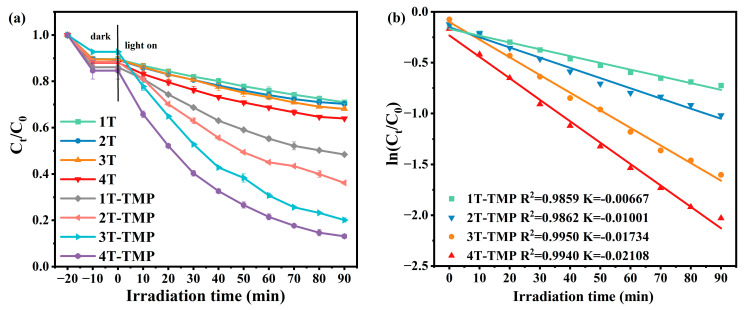
(**a**) Photocatalytic degradation efficiency of MB by TiO_2_ and TiO_2_-TMP and (**b**) fitting results of photocatalytic degradation kinetics of TiO_2_-TMP.

The photocatalytic activity of TiO_2_-TMP was evaluated by degrading MB in visible light, and the influence of TiO_2_ crystal form on the photocatalytic performance of TiO_2_ under visible light was studied. Firstly, an adsorption experiment was conducted under dark conditions for 20 min (Figure 13a). After adsorption, TiO_2_ and TiO_2_-TMP only slightly degraded MB. As shown in Figure 13a, after 90 min of visible light irradiation, the degradation rates of 1T-TMP, 2T-TMP, 3T-TMP, and 4T-TMP were 51.55%, 63.85%, 79.87%, and 86.87%, respectively. All TiO_2_-TMP composites exhibited a better photocatalytic performance than TiO_2_. Meanwhile, the kinetic behavior of MB degradation was further studied using the Langmuir–Hinshelwood model. The experimental results were fitted using the pseudo-first-order kinetic equation, as shown in Equation (8) [66], as follows:

ln(C_t_/C_0_) = −K_app_ t (8)

where C_0_ (mg/L) is the initial concentration of MB, C_t_ (mg/L) is the concentration of MB during the photocatalytic degradation process for t min, and K_app_ (min^−1^) is the pseudo-first-order kinetic rate constant. Figure 13b shows that 4T-TMP (K_app_ = 0.02108 min^−1^) showed a higher apparent rate constant. It was notable that the photocatalytic degradation rate and K_app_ value of TiO_2_-TMP composites gradually increased with an increase in anatase phase content. This was consistent with the variation trends of the interaction at the TiO_2_-TMP interface, the BIEF strength of the heterojunction, and the specific surface area. The above results indicate that the TiO_2_-TMP composite material exhibited more excellent photocatalytic activity than TiO_2_ under visible light. This is because the strong interface interaction and heterojunction BIEF can enhance the transfer and separation efficiency of photogenerated electrons and holes; a higher specific surface area and abundant pore structure provide more active catalytic sites, thereby increasing the degradation rate of MB.

#### 3.4.2. Analysis of Active Free Radicals

**Figure 14:**
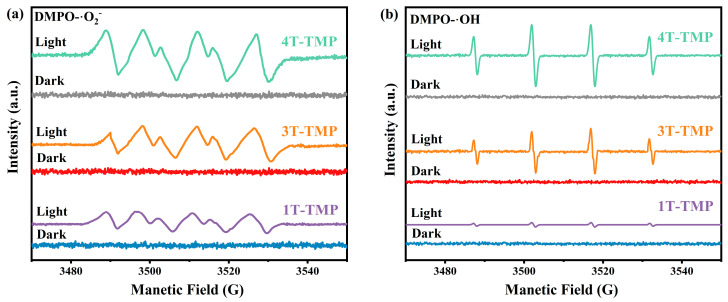
(**a**) ESR signal intensity of •O_2_^−^ radicals in 1T-TMP, 3T-TMP, and 4T-TMP materials under light and darkness and (**b**) ESR signal intensity of •OH radicals in 1T-TMP, 3T-TMP, and 4T-TMP materials under light and darkness.

To explore the mechanism of photocatalytic degradation, a series of free radical capture experiments were carried out. It can be found from Figure S10a,b that after adding AgNO_3_, the photocatalytic degradation efficiency changed slightly compared with the system without scavengers, indicating that e- was not the main active species in the MB degradation process. Conversely, after the addition of MeOH, pBQ, and EDTA-2Na, the photocatalytic performance decreased significantly, indicating that •OH, •O_2_^−^, and h^+^ were the main active species. Meanwhile, ESR characterization was carried out (Figure 14a,b). Under the condition of no light, no signals of •OH and •O_2_^−^ were detected in 1T-TMP, 2T-TMP, 3T-TMP, and 4T-TMP. However, after 10 min of light exposure, both could be detected. Furthermore, as the content of the anatase phase increased, the signal strength of the TiO_2_-TMP composite material gradually strengthened. This indicates that more •OH and •O_2_^−^ free radicals were generated in the 4T-TMP composite material, corresponding to a better photocatalytic degradation effect. Therefore, for the TiO_2_-TMP composite material, the degradation sequence of MB by each active species was •O_2_^−^ > h^+^ > •OH.

#### 3.4.3. Analysis of Photocatalytic Mechanism

**Figure 15:**
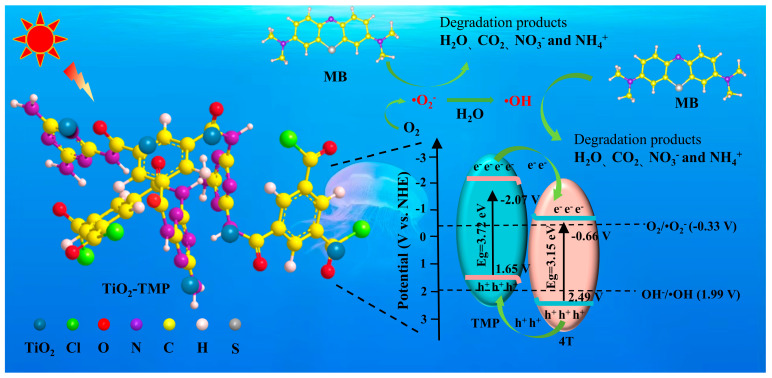
Degradation mechanism diagram of TiO_2_-TMP.

Based on the above analysis, the mechanism of MB degradation by TiO_2_-TMP photocatalysts is proposed (Figure 15). The Ti-O-N bond formed by TiO_2_ and TMP provides an electron transport channel. The potential difference between TiO_2_ and TMP forms the BIEF from TiO_2_ to TMP. Driven by BIEF, the photogenerated electrons enriched in the HOMO energy level of TMP are transferred to the CB of TiO_2_, and holes on the VB of TiO_2_ are transferred to the HOMO energy level of TMP. This Type II heterojunction charge transfer mechanism realizes the spatial separation and efficient utilization of photogenerated carriers. The electrons on the conduction band of TiO_2_ can react with O_2_ to form •O_2_^−^. The holes on the VB of TMP cannot produce •OH by oxidizing H_2_O and OH^−^. However, •O_2_^−^ can be rapidly converted to •OH in aqueous solution. Therefore, a part of the •O_2_^−^ is directly used to attack pollutants, and a part is converted into •OH. Under the attack of •O_2_^−^, h^+^, and •OH, MB is decomposed into small molecules and further mineralized into CO_2_, H_2_O, NO_3_^−^, and NH_4_^+^.

## 4. Conclusions

In conclusion, in this paper, the photocatalytic activities of different crystal forms of TiO_2_-TMP composites were comprehensively evaluated through XRD, BET, TEM, FT-IR, XPS, UV-vis DR, UPS characterization, DFT theoretical calculation, and the photocatalytic degradation of MB. The research found that the particle size, specific surface area, crystal plane characteristics, surface hydroxyl concentration, electronic structure, and atomic arrangement of titanium dioxide affect the interfacial contact between titanium dioxide and TMP and the content of Ti-O-N bonds. The band structure of TiO_2_ affects the formation of TiO_2_-TMP heterojunctions. With an increase in anatase content, the interfacial contact between TiO_2_ and TMP is enhanced, the content of Ti-O-N bonds increases, and the built-in electric field strength of the heterojunction increases. Strong interfacial interactions and built-in electric fields can enhance the transfer and separation efficiency of electrons and holes. A larger surface area and pore volume can provide more active sites for photocatalytic reactions, thereby increasing the degradation rate of MB. This work provides certain references and guidance for the design of new heterojunction photocatalysts and the explanation of the interface interaction mechanism of organic–inorganic composites.

## Data Availability

The original contributions presented in this study are included in the article/Supplementary Material. Further inquiries can be directed to the corresponding authors.

## Supplementary Materials

The following supporting information can be downloaded at: https://www.mdpi.com/article/10.3390/ma18112623/s1, Refs. [67,68,69,70,71,72,73,74,75,76,77,78] are cited in the Supplementary Materials. Figure S1: (**a**) SEM and (**b**) TEM images of 1T; (**c**) SEM and (**d**) TEM images of 1T-TMP; (**e**) SAED patterns of 1T-TMP; (**f**) HRTEM images of 1T-TMP; (**g**) EDS mapping images of 1T-TMP; Figure S2: FTIR spectra of TiO_2_; Figure S3: (**a**) XPS spectra of C 1s in TMP and 1T-TMP; (**b**) XPS spectra of O 1s in 1T and 1T-TMP; (**c**) XPS spectra of Ti 2p in 1T and 1T-TMP; Figure S4: (**a**) XPS spectra of C 1s in TMP and 2T-TMP; (**b**) XPS spectra of O 1s in 2T and 2T-TMP; (**c**) XPS spectra of Ti 2p in 2T and 2T-TMP; Figure S5: (**a**) XPS spectra of C 1s in TMP and 3T-TMP; (**b**) XPS spectra of O 1s in 3T and 3T-TMP; (**c**) XPS spectra of Ti 2p in 3T and 3T-TMP; Figure S6: (**a**) UV-Vis DRS spectra of TiO_2_ and TiO_2_-TMP; (**b**) Diagram of the Kubelka–Munk function of the TiO_2_ and TMP versus the absorbed light energy; (**c**) Cyclic voltammogram (CV) of TMP; Figure S7: (**a**) Open-circuit potentials of TiO_2_-TMP; (**b**) surface charge densities of TiO_2_-TMP; Figure S8: (**a**) The average charge density difference of anatase phase TiO_2_ (101)-C_3_N_3_H_3_; (**b**) The average charge density difference of brookite phase TiO_2_ (121)-C_3_N_3_H_3_; Figure S9: The projected density of states of Ti atoms of anatase phase TiO_2_ (101) and brookite phase TiO_2_ (121); Figure S10: (**a**) The photocatalytic removal curve of MB in the free radical capture experiment; (**b**) The photocatalytic removal efficiency of MB in the free radical capture experiment; Figure S11: Cyclic experiments of (**a**) 4T-TMP; (**c**) 3T-TMP; (**e**) 2T-TMP; (**g**) 1T-TMP for solving MB in visible light degradation; XRD patterns before and after photodegradation of (**b**) 4T-TMP; (**d**) 3T-TMP; (**f**) 2T-TMP; (**h**) 1T-TMP; Table S1: Phase contents and crystalline sizes of TiO_2_ and TiO_2_-TMP; Table S2: Surface area and pore volume of TiO_2_ and TiO_2_-TMP; Table S3: Chemical bond content in TiO_2_-TMP based on XPS data; Table S4: Comparison of adsorption energy between anatase (101) and brookite (121) crystal plane.
