# Supplementary material for: Preparation and Photocatalytic Performance Study of TiO2-TMP Composites Under Effect of Crystal Structure Modulation
Source: Materials (Basel). 2025 Jun 3;18(11):2623. doi: 10.3390/ma18112623 (PMC12156310; doi:10.3390/ma18112623)
Supplement: Supplementary file 1 [file materials-18-02623-s001.zip › materials-3649717-supplementary.pdf]

materials

MDPI

Supplementary Materials

Preparation and Photocatalytic Performance Study of TiO_2-TMP Composites under Effect of Crystal Structure Modulation

Text S1. Calculation of the built-in electric field intensity (BIEF)

We calculated the BIEF of TiO_2-TMP using the model (Equation (1)) developed by Kanat et al [1].

$$Fs = (-2Vs\varrho/\varepsilon\varepsilon_0)^{1/2} \quad (1)$$

Copyright: © 2025 by the authors. Licensee MDPI, Basel, Switzerland. This article is an open access article distributed under the terms and conditions of the Creative Commons Attribution (CC BY) license (https://creativecommons.org/licenses/by/4.0/).

where Fs is the BIEF magnitude, Vs is the surface voltage, ϱ is the surface charge density, ε is the low-frequency dielectric constant, and ε_0 is the free-space dielectric constant. Since ε and ε_0 are two constants, the magnitude of BIEF is mainly determined by the surface voltage and charge density. To evaluate the variation of the BIEF magnitude of the sample, we carefully calculated the surface voltage of the sample through open-circuit potential measurement [2-4]. The charge density was calculated simultaneously by potentiometric titration and conductance titration [2–4].

Text S2. Electrochemical impedance spectroscopy (EIS), transient photocurrent density (It) and cyclic voltammetry (CV) characterization

The electrochemical impedance spectroscopy, transient photocurrent density and cyclic voltammetry were obtained by CHI-660E electrochemical workstation (CH Instruments, Shanghai, China), and the working electrochemical, counter electrode, and reference electrode are ITO electrode, platinum electrode and saturated Ag/AgCl electrode, respectively. The preparation method of the working electrode: 10 mg of the sample, 0.01 mL of Nafion solution and 0.09 ml of anhydrous ethanol were mixed together and ultrasonically treated for 30 minutes to form a uniform suspension. Afterwards, the solution was coated onto the 1×1 cm^2 ITO conductive glass, and allowed to dry naturally at room temperature. In the photocurrent experiment, Na_2SO_4 solution (0.1 M) was used as the electrolyte, and a 300W xenon lamp was employed as the light source. The experiment was conducted by turning on/off the lamp every 50 seconds. EIS is determined over the frequency range of 10^2 - 10^6 Hz with an ac amplitude of 10 mV at the open circuit voltage under room-light illumination. Furthermore, the cyclic voltammetry method is used to calculate the position of the TMP polymer band gap. Ferrocene/ferrocenium (Fc/Fc^+) as an internal standard system, and 0.1 M $Bu_4N_PF_6$ solution as the electrolyte. The oxidation peak potential $E_{Fc/Fc+}$ of ferrocene/ferrocenium (Fc/Fc^+) relative to the Ag/AgCl reference electrode was determined to be 0.29 V through cyclic voltammetry test, and the absolute potential reference value of this system under vacuum conditions was 4.8 eV vs. Ag/AgCl. The highest occupied molecular orbital (HOMO) energy level is calculated based on the formula $E_{HOMO} = -(E_{OX} + 4.8 - E_{Fc/Fc+})$, where E_{OX} represents the oxidation initiation potential of the material itself [5]. Then calculate the LUMO energy level based on the equation $E_{LUMO} = E_{HOMO} + Eg$.

 https://doi.org/10.3390/ma18112623

Text S3. Free radical capture experiment

Methanol (MeOH), p-benzoquinone (pBQ), silver nitrate ($AgNO_3$) and ethylenediaminetetraacetic acid (EDTA-2Na) were selected as inhibitors of hydroxyl radicals (•OH), superoxide radicals ($•O_2^-$), photogenerated electrons (e^-) and photogenerated holes (h^+), respectively. Under the conditions of the same irradiation intensity and catalyst dosage, the quencher was introduced. After the photocatalytic degradation of MB reaction was completed, the contribution rate of different active species to pollutants was quantitatively evaluated by comparing the degradation efficiency of the blank group and the quenching group. Its specific operation process is consistent with the process of photocatalytic degradation of MB.

Text S4. Theoretical calculation methods

Density functional theory (DFT) calculations were conducted using the Vienna Ab initio Simulation Package (VASP) [6]. The Perdew-Burke-Ernzerhof (PBE) functional in Generalized Gradient Approximation (GGA) is adopted to describe the exchange correlation interaction [7]. The plane-wave energy cutoff was set to 450 eV to ensure convergence of the wavefunctions and maintain computational precision. The electronic self-consistent field (SCF) convergence criterion was set to 1×10^{-6} eV, and the ionic relaxation force convergence criterion was set to 0.03 eV/Å, ensuring numerical stability and high accuracy of the results. A Γ-centered (1×1×1) k-point grid was utilized, appropriate for small systems. To account for van der Waals interactions, the DFT-D3 dispersion correction method developed by Grimme et al. was applied [8].

In the previous DFT research calculations, the (101) crystal plane was the low-energy plane of the anatase phase, and the (121) crystal plane was the low-energy plane of the brookite phase [9], which were the most stable surfaces. Under natural conditions, crystals preferentially grow along the direction with high surface energy, and eventually expose the crystal plane with the lowest surface energy [10, 11]. Therefore, in the TiO_2 structure simulation, the anatase phase (101) crystal plane and the brookite phase (121) crystal plane are usually selected as typical exposed planes. TMP is a highly cross-linked structure, formed by multiple monomers connected through covalent bonds to create organic compounds with higher molecular weights, and its model is rather difficult to construct. The research found that the TMP structure can be simulated by truncating the reaction site [12].

Table S1. Phase contents and crystalline sizes of TiO_2 and TiO_2-TMP.

Material	[1] Phase content fraction (wt.%)		[2] Crystallite Size(nm)	
	A	B	A	B
1T	0	100	—	49.31
2T	34.7	65.3	38.37	40.56
3T	69.8	30.2	30.49	39.16
4T	100	0	45.52	—
1T-TMP	0	100	—	36.32
2T-TMP	24.7	75.3	28.12	32.09
3T-TMP	69.8	30.2	20.15	37.85
4T-TMP	100	0	34.52	—

[1] Calculate anatase and brookite phase content according to Scherrer formula: $W_A = K_AI_A / (K_AI_A + K_BI_B)$, $W_B = K_BI_B (K_AI_A + K_BI_B)$. WA and WB are the components of anatase and brookite, respectively. IA and IB are the comprehensive intensity of anatase (101) and brookite (121) diffraction peaks, respectively. Optimization coefficient KA = 0.886, KB = 2.217.

[2] Calculate crystal size according to Debye-Scherrer formula: $d = 0.89\lambda / (\beta \times \cos \theta)$, where $\lambda = 0.154$ nm, θ is the Bragg angle, and β is the full wide at half the maximum intensity (FWHM) for the main peaks (101) and (121) for anatase phase and brookite phase, respectively.

Table S2. Surface area and pore volume of TiO_2 and TiO_2-TMP.

Material	Surface area (m^2g^{-1})	Pore volume (cm^3g^{-1})
1T	8.558	0.022
2T	9.55	0.035
3T	11.132	0.048
4T	13.464	0.056
1T-TMP	10.616	0.034
2T-TMP	12.373	0.042
3T-TMP	13.98	0.069
4T-TMP	16.41	0.072

Table S3. Chemical bond content in TiO_2-TMP based on XPS data.

Sample	1T-TMP	2T-TMP	3T-TMP	4T-TMP
C 1s				
C-C	67.18%	78.52%	76.77%	62.45%
C-O	24.29%	13.42%	7.56%	15.71%
Ti-O-C	6.52%	8.06%	15.66%	21.85%
O 1s				
Ti-O	77.01%	66.24%	62.74%	47.50%
Ti-O-N	15.67%	26.32%	32.12%	49.08%
C-O	7.32%	7.44%	5.14%	3.43%

Table S4. Comparison of adsorption energy between anatase (101) and brookite (121) crystal plane.

**Adsorbed crystal plane**	**Adsorption energy (eV)**
anatase (101)	-1.63
brookite (121)	0.016

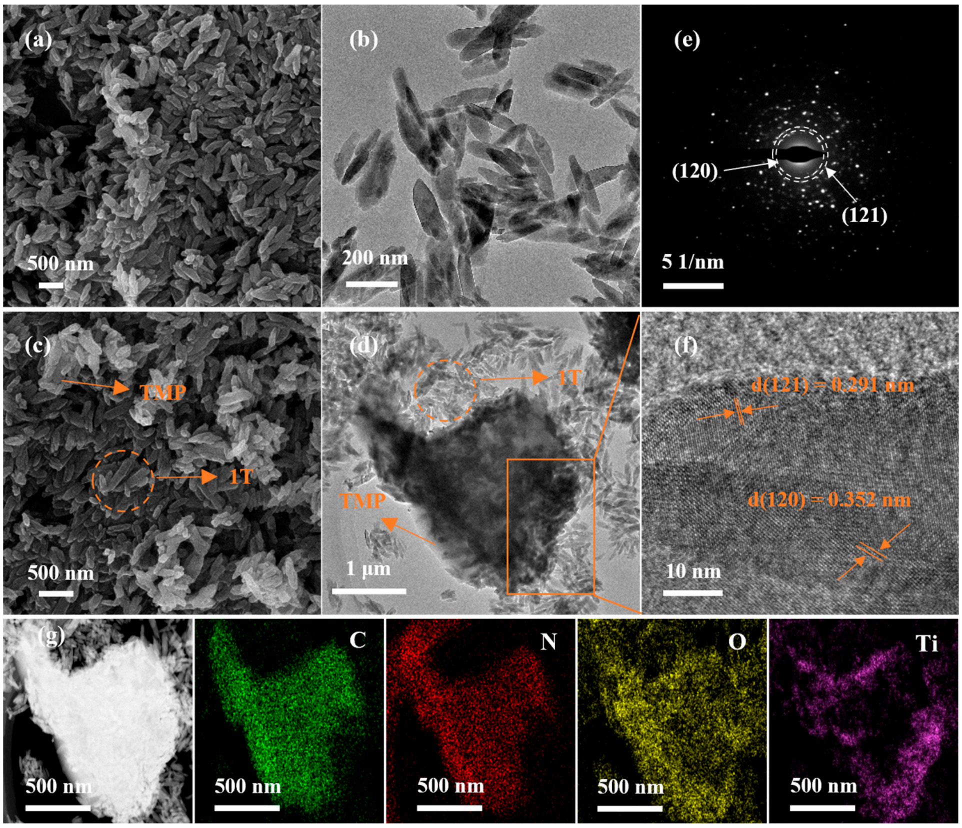

Figure S1. (**a**) SEM and (**b**) TEM images of 1T; (**c**) SEM and (**d**) TEM images of 1T-TMP; (**e**) SAED patterns of 1T-TMP; (**f**) HRTEM images of 1T-TMP; (**g**) EDS mapping images of 1T-TMP.

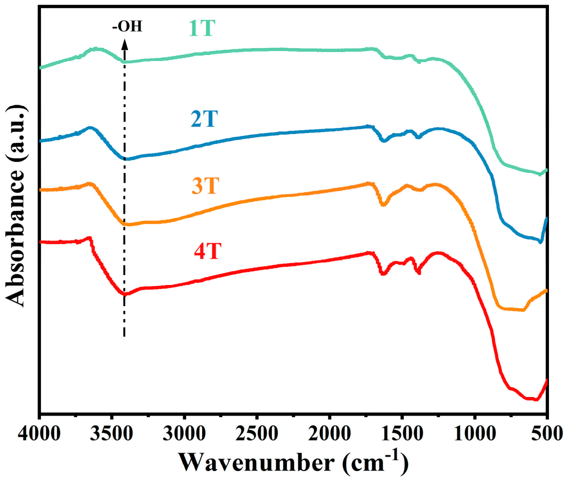

Figure S2. FTIR spectra of TiO_2

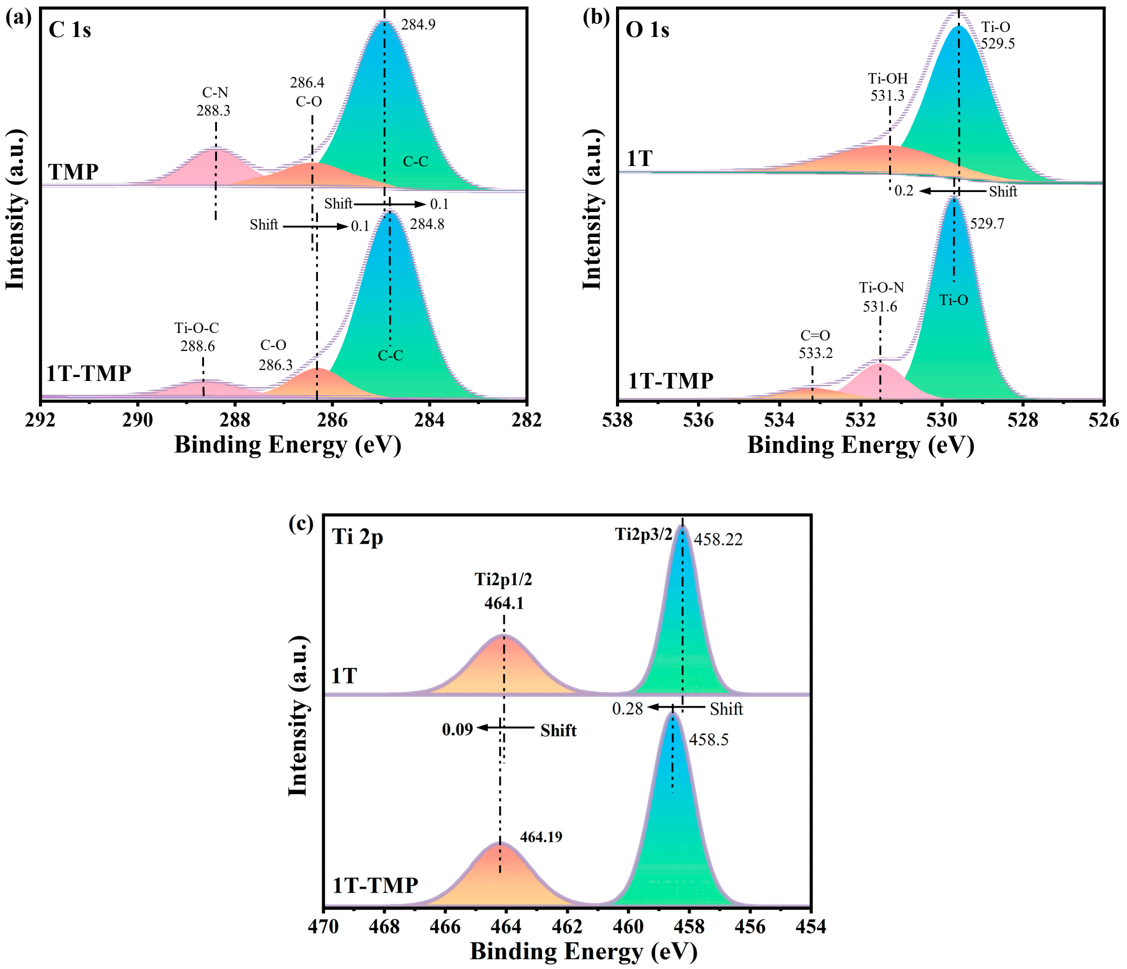

Figure S3. (**a**) XPS spectra of C 1s in TMP and 1T-TMP; (**b**) XPS spectra of O 1s in 1T and 1T-TMP; (**c**) XPS spectra of Ti 2p in 1T and 1T-TMP.

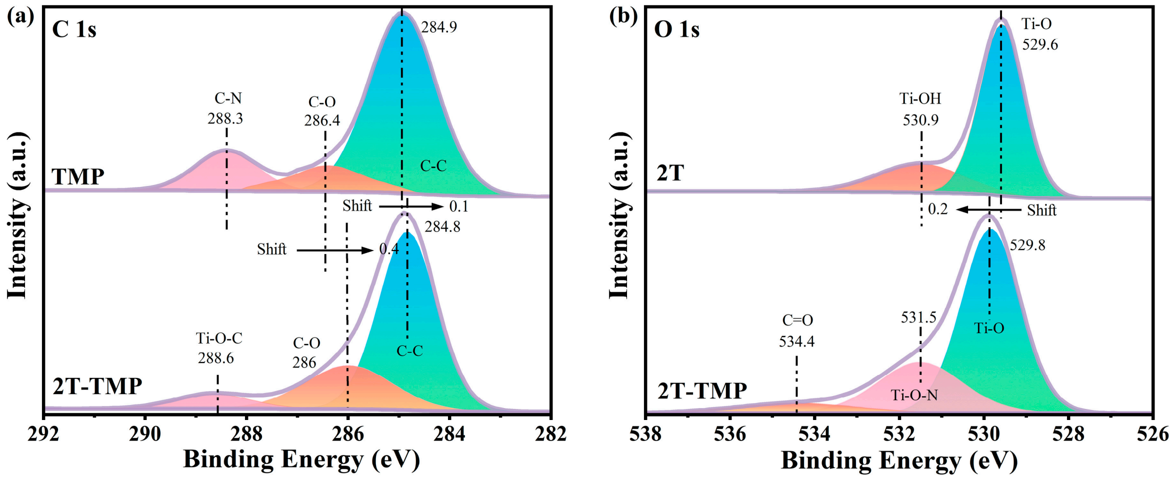

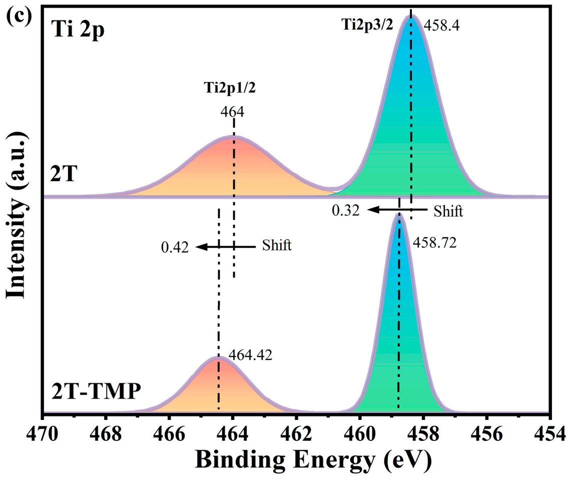

Figure S4. (**a**) XPS spectra of C 1s in TMP and 2T-TMP; (**b**) XPS spectra of O 1s in 2T and 2T-TMP; (**c**) XPS spectra of Ti 2p in 2T and 2T-TMP.

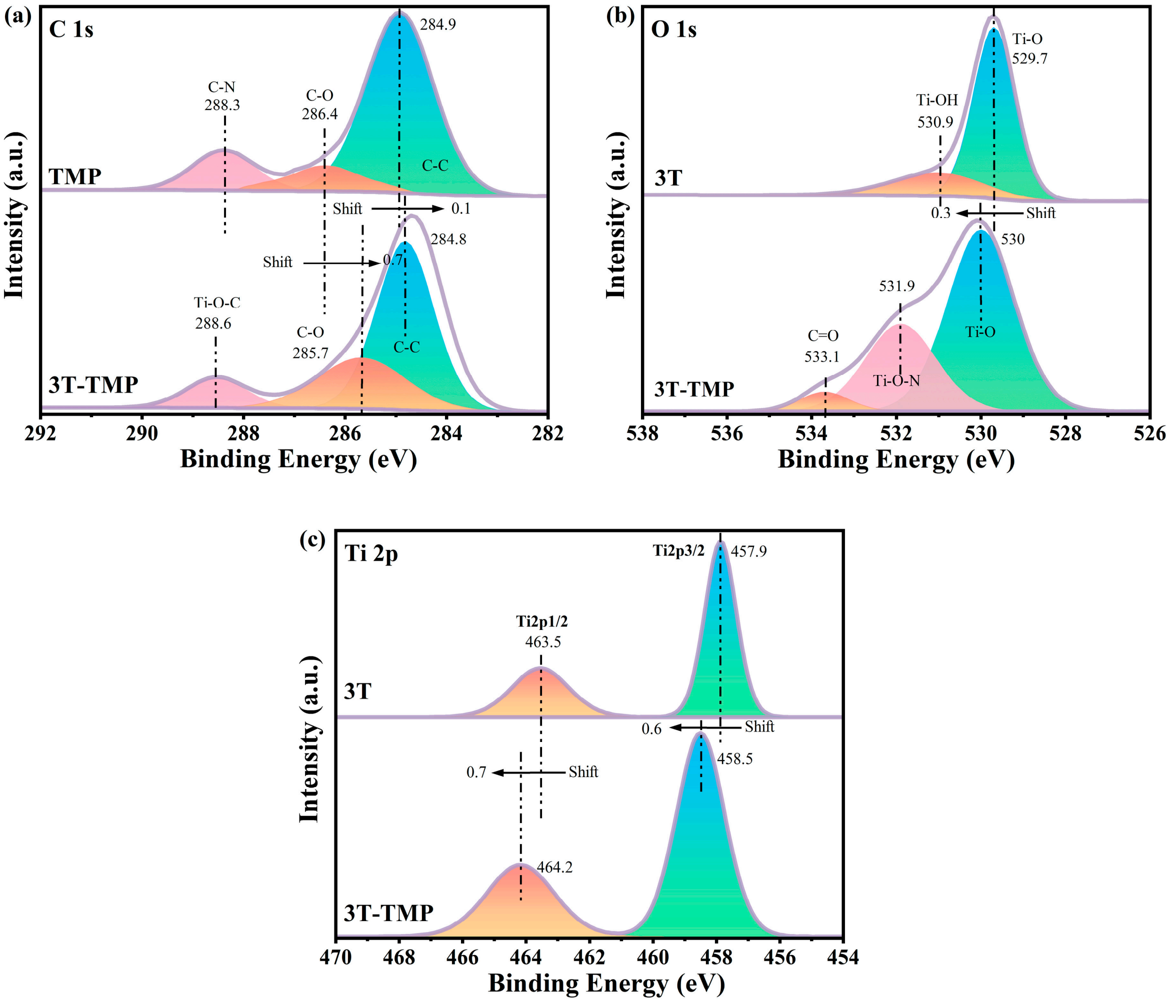

Figure S5. (**a**) XPS spectra of C 1s in TMP and 3T-TMP; (**b**) XPS spectra of O 1s in 3T and 3T-TMP; (**c**) XPS spectra of Ti 2p in 3T and 3T-TMP.

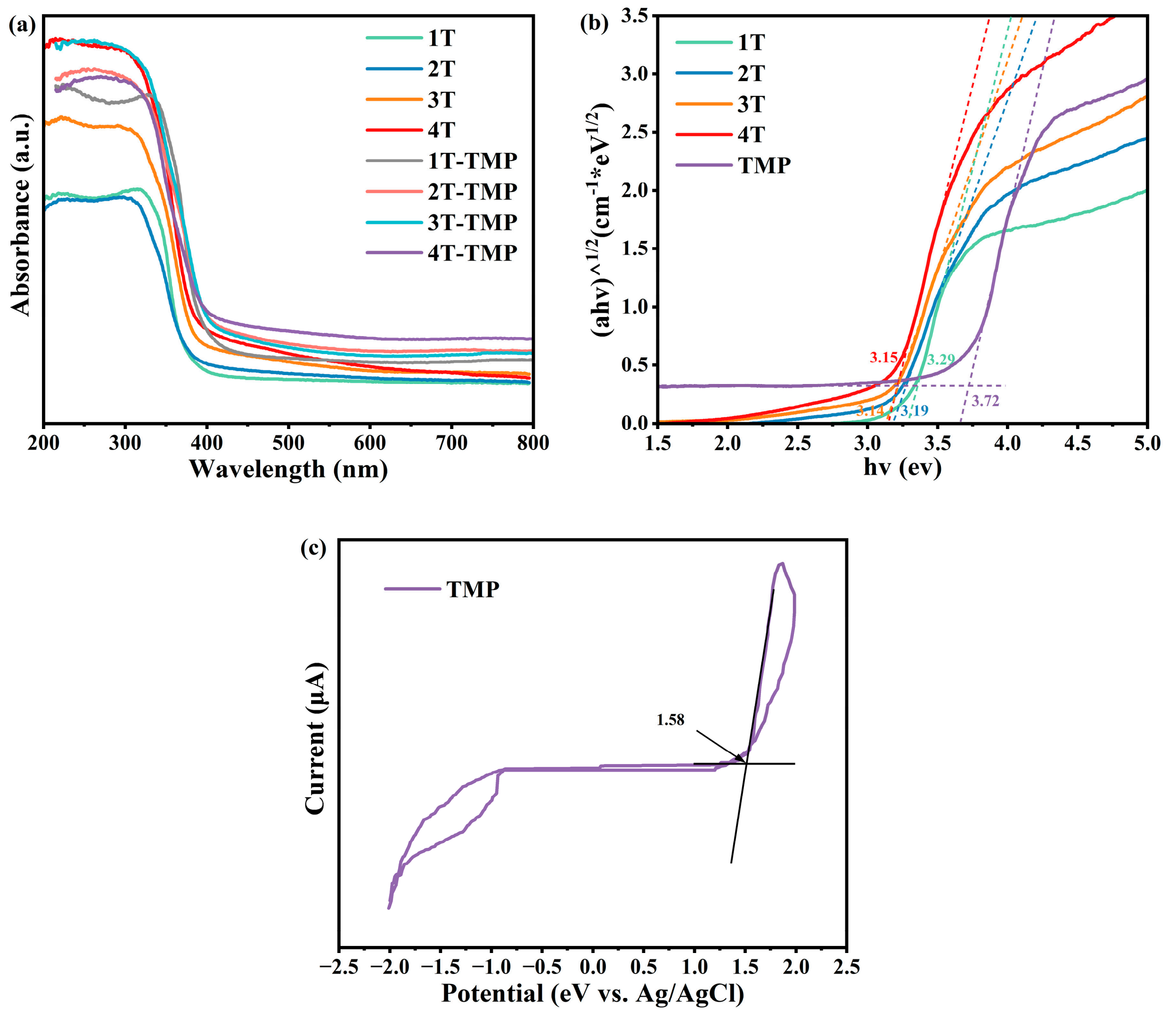

Figure S6. (**a**) UV-Vis DRS spectra of TiO_2 and TiO_2-TMP; (**b**) Diagram of the Kubelka–Munk function of the TiO_2 and TMP versus the absorbed light energy; (**c**) Cyclic voltammogram (CV) of TMP.

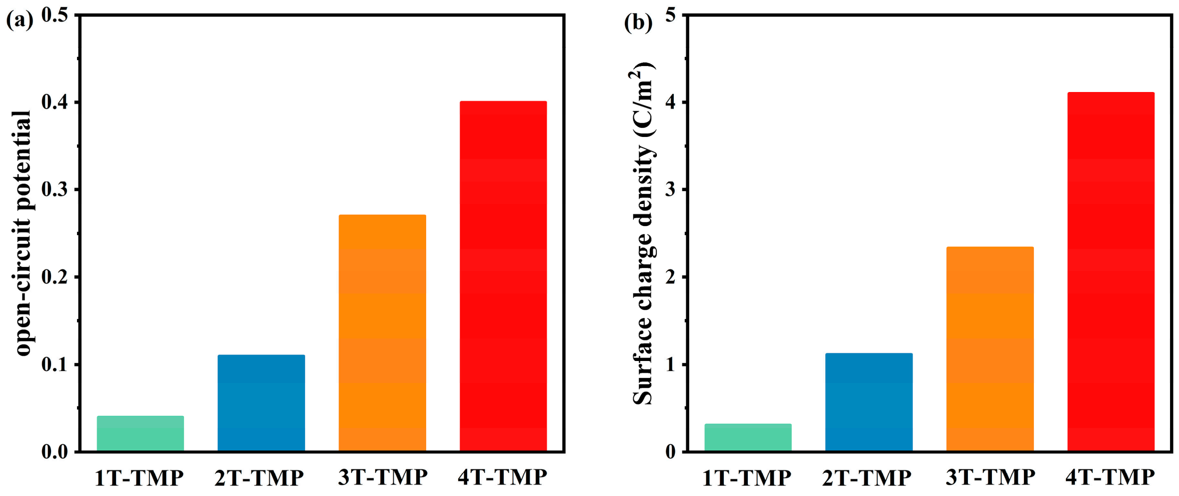

Figure S7. (**a**) Open-circuit potentials of TiO_2-TMP; (**b**) surface charge densities of TiO_2-TMP.

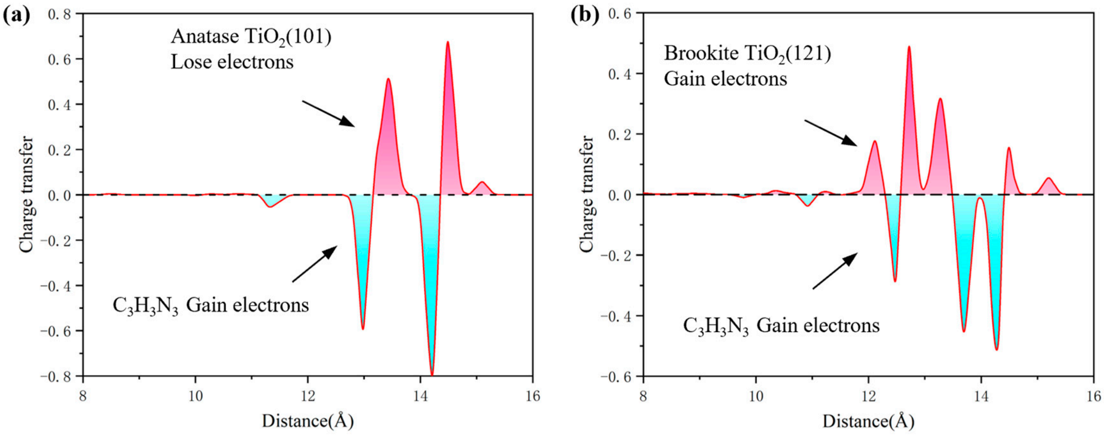

Figure S8. (**a**) The average charge density difference of anatase phase TiO_2 (101)-$C_3N_3H_3$; (**b**) The average charge density difference of brookite phase TiO_2 (121)-$C_3N_3H_3$.

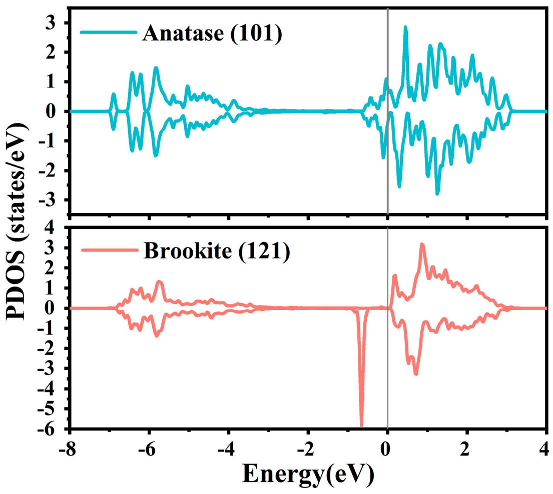

Figure S9. The projected density of states of Ti atoms of anatase phase TiO_2 (101) and brookite phase TiO_2 (121).

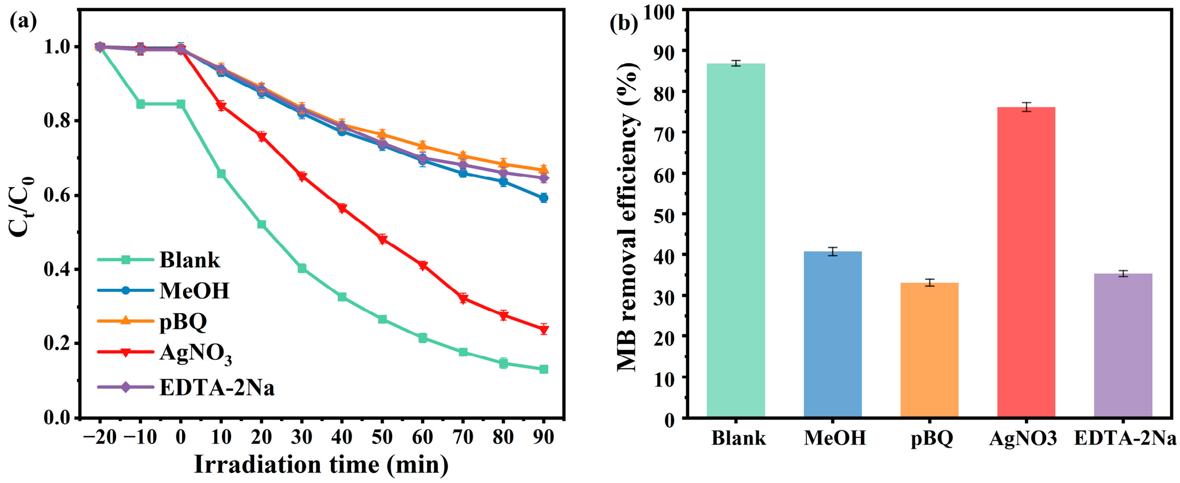

Figure S10. (**a**) The photocatalytic removal curve of MB in the free radical capture experiment; (**b**) The photocatalytic removal efficiency of MB in the free radical capture experiment.

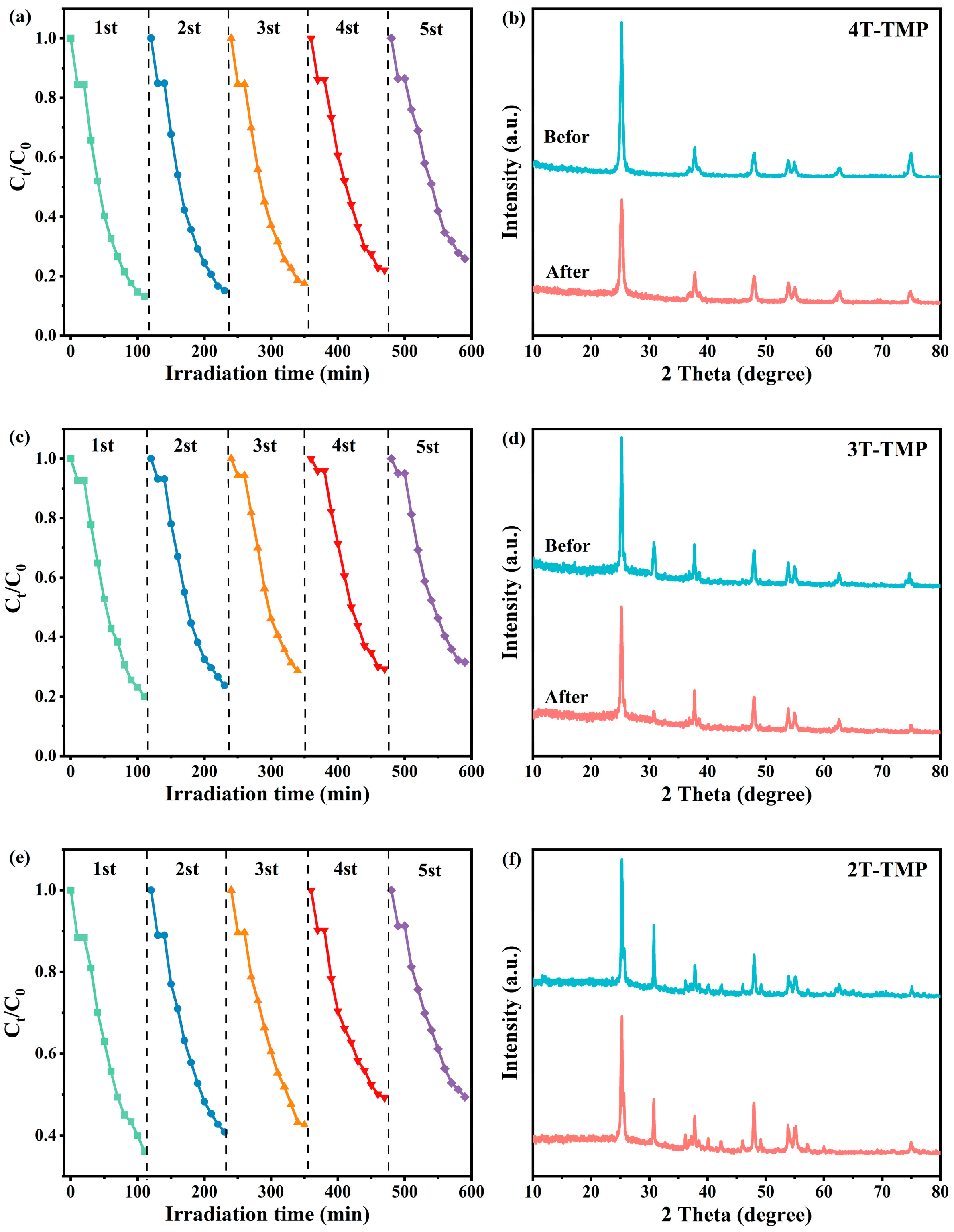
(a)
1st
2st
3st
4st
5st
Ct/C0
Irradiation time (min)
(b)
4T-TMP
Befor
After
Intensity (a.u.)
2 Theta (degree)
(c)
1st
2st
3st
4st
5st
Ct/C0
Irradiation time (min)
(d)
3T-TMP
Befor
After
Intensity (a.u.)
2 Theta (degree)
(e)
1st
2st
3st
4st
5st
Ct/C0
Irradiation time (min)
(f)
2T-TMP
Intensity (a.u.)
2 Theta (degree)

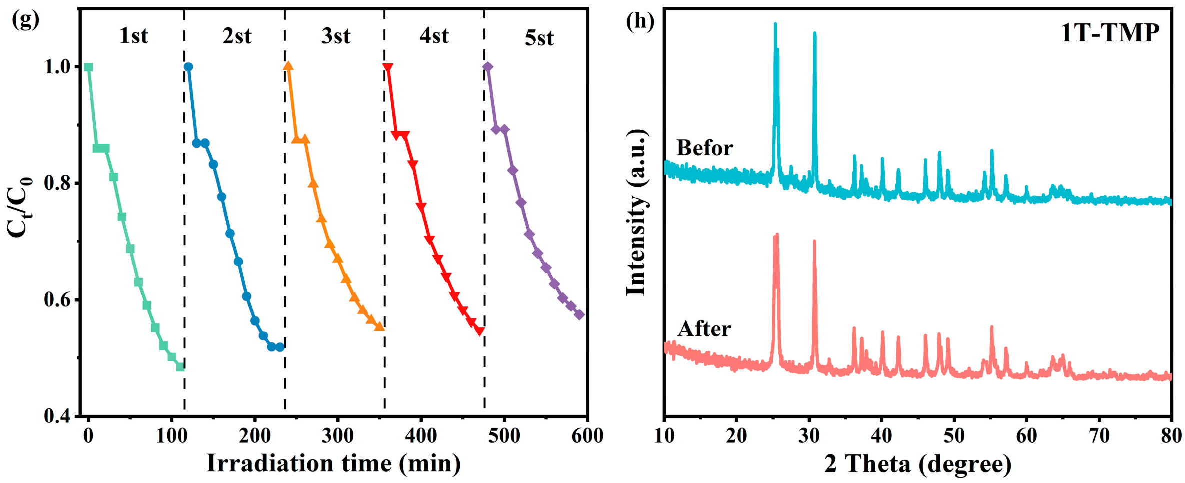

Figure S11. Cyclic experiments of (**a**) 4T-TMP; (**c**) 3T-TMP; (**e**) 2T-TMP; (**g**) 1T-TMP for solving MB in visible light degradation; XRD patterns before and after photodegradation of (**b**) 4T-TMP; (**d**) 3T-TMP; (**f**) 2T-TMP; (**h**) 1T-TMP.

Considering the possibility of practical application, the cycling degradation rate of the TiO_2-TMP photocatalytic system was studied. As shown in Figure S9a, c, e, g, after five consecutive photocatalytic degradations of MB, 4T-TMP, 3T-TMP, 2T-TMP and 1T-TMP still demonstrated high activity. Compared with the first time, the removal rate only decreased by 12.74%, 11.5%, 13.23% and 9.04% respectively. Furthermore, by comparing the XRD patterns before and after degradation (Figure S9b, d, f, h), it was observed that the intensity of the characteristic peaks slightly weakened, but the positions of most characteristic peaks remained unchanged. The above results fully proved that the prepared 4T-TMP composite material has good cycling stability and practicability.

References

1. Lefebvre, P.; Allègre, J.; Gil, B.; Mathieu, H.; Grandjean, N.; Leroux, M.; Massies, J.; Bigenwald, P. Time-resolved photoluminescence as a probe of internal electric fields in GaN-(GaAl)N quantum wells. *Physical Review B.* **1999,** *59*, 15363-15367. http://doi.org/10.1103/PhysRevB.59.15363
2. Yanina, S. V.; Rosso, K. M. Linked Reactivity at Mineral-Water Interfaces Through Bulk Crystal Conduction. *Science.* **2008,** *320*, 218-222. http://doi.org/10.1126/science.1154833
3. Prado, A. G. S.; Bolzon, L. B.; Pedroso, C. P.; Moura, A. O.; Costa, L. L. Nb_2O_5 as efficient and recyclable photocatalyst for indigo carmine degradation. *Applied Catalysis B: Environmental.* **2008,** *82*, 219-224. http://doi.org/https://doi.org/10.1016/j.apcatb.2008.01.024
4. Kallay, N.; Madić, T.; Kučej, K.; Preočanin, T. Enthalpy of interfacial reactions at TiO_2 aqueous interface. *Colloids and Surfaces A: Physicochemical and Engineering Aspects.* **2003,** *230*, 3-11. http://doi.org/https://doi.org/10.1016/j.colsurfa.2003.09.008
5. Yu, F.; Zhu, Z.; Li, C.; Li, W.; Liang, R.; Yu, S.; Xu, Z.; Song, F.; Ren, Q.; Zhang, Z. A redox-active perylene-anthraquinone donor-acceptor conjugated microporous polymer with an unusual electron delocalization channel for photocatalytic reduction of uranium (VI) in strongly acidic solution. *Applied Catalysis B: Environmental.* **2022,** *314*. http://doi.org/10.1016/j.apcatb.2022.121467
6. Kresse, G.; Furthmüller, J. Efficient iterative schemes for ab initio total-energy calculations using a plane-wave basis set. *Physical Review B.* **1996,** *54*, 11169-11186. http://doi.org/10.1103/PhysRevB.54.11169
7. Perdew, J. P.; Burke, K.; Ernzerhof, M. Generalized Gradient Approximation Made Simple. *Physical Review Letters.* **1996,** *77*, 3865-3868. http://doi.org/10.1103/PhysRevLett.77.3865
8. Grimme, S.; Ehrlich, S.; Goerigk, L. Effect of the damping function in dispersion corrected density functional theory. *Journal of Computational Chemistry.* **2011,** *32*, 1456-1465. http://doi.org/10.1002/jcc.21759

9. Gong, X.-Q.; Selloni, A. First-principles study of the structures and energetics of stoichiometric brookite TiO_2 surfaces. *Physical Review B.* **2007,** *76*, 235307. http://doi.org/10.1103/PhysRevB.76.235307
10. Gao, Z.-y.; Sun, W.; Hu, Y.-h.; Liu, X.-w. Surface energies and appearances of commonly exposed surfaces of scheelite crystal. *Transactions of Nonferrous Metals Society of China.* **2013,** *23*, 2147-2152. http://doi.org/10.1016/s1003-6326(13)62710-7
11. Shah, U. V.; Olusanmi, D.; Narang, A. S.; Hussain, M. A.; Gamble, J. F.; Tobyn, M. J.; Heng, J. Y. Y. Effect of crystal habits on the surface energy and cohesion of crystalline powders. *International Journal of Pharmaceutics.* **2014,** *472,* 140-147. http://doi.org/10.1016/j.ijpharm.2014.06.014
12. Hall, S. A.; Howlin, B. J.; Hamerton, I.; Baidak, A.; Billaud, C.; Ward, S. Solving the Problem of Building Models of Crosslinked Polymers: An Example Focussing on Validation of the Properties of Crosslinked Epoxy Resins. *PLOS ONE.* **2012,** *7,* e42928. http://doi.org/10.1371/journal.pone.0042928

Disclaimer/Publisher's Note: The statements, opinions and data contained in all publications are solely those of the individual author(s) and contributor(s) and not of MDPI and/or the editor(s). MDPI and/or the editor(s) disclaim responsibility for any injury to people or property resulting from any ideas, methods, instructions or products referred to in the content.
